# Secret shopper studies: an unorthodox design that measures inequities in healthcare access

**DOI:** 10.1186/s13690-022-00979-z

**Published:** 2022-11-04

**Authors:** Kelsey A. Rankin, Alison Mosier-Mills, Walter Hsiang, Daniel H. Wiznia

**Affiliations:** Yale School of Medicine, 333 Cedar Street, 06510 New Haven, CT USA

**Keywords:** Secret shopper, Healthcare delivery, Methodology, Phone interviews, Accessibility

## Abstract

Secret shopper studies are particularly potent study designs that allow for the gathering of objective data for a variety of research hypotheses, including but not limited to, healthcare delivery, equity of healthcare, and potential barriers to care. Of particular interest during the COVID-19 pandemic, secret shopper study designs allow for the gathering of data over the phone. However, there is a dearth of literature available on appropriate methodological practices for these types of studies. To make these study designs more widely accessible, here we outline the case for using the secret shopper methodology and detail best practices for designing and implementing them.

## Background

The secret shopper study design—also commonly referred to as “mystery shoppers” or “simulated patients”—is an integral study technique that allows for the exploration of multiple parameters of healthcare delivery. The strength of the secret shopper study design is that it provides insight into the challenges of access to healthcare that may be difficult to measure through more standard investigative techniques. By contacting healthcare facilities and posing as a patient seeking care, secret shopper investigators can attain a realistic, and more importantly, unbiased perspective of the patient experience [1]. This methodology is useful across many disciplines, as it is broadly relevant and well-suited for real-time quality improvement/preventative strategies. For example, in disciplines such as family medicine, these studies can illuminate issues in the healthcare pathway stemming from appointment scheduling, patient experience with various administrative staff and/or healthcare providers, accessibility to specialty care, differential treatment based on race, gender, sexuality, disability, age, anthropomorphic metrics, etc., and insurance status [2–5].

Our group believes strongly in the secret shopper research design, and we have successfully designed, implemented, and published over 20 secret shopper studies evaluating patient access to care, primarily probing the effect of patient insurance status. We looked to landmark studies that have utilized secret shopper techniques when developing our protocols, drafting our call scripts, and presenting our data. However, we noted a dearth of methodological literature available for this study design, most prominently for studies that employed phone calls rather than in-person simulated patient visits. This was of particular note in 2021, as the COVID-19 pandemic has changed the ways in which we deliver healthcare with a newfound focus on telemedicine. Telemedicine has shown us the scope of the interactions providers and patients are able to have via the telephone or video chat. Patients are able to have satisfying interactions with their providers, but this new landscape merits assessment of the quality and equity of the care provided. Secret shopper study designs have significant potential in providing unbiased assessments of care provided via telemedicine, but we strongly caution against actual care provision in the study design methodology. Here we outline the case for using the secret shopper methodology and detail best practices for designing and implementing them. We also include a case study analysis to describe how our research navigated the unique challenges inherent to the secret shopper methodology.

## Methods

### Data sources and collection mode

Investigators can use simulated patients to collect data via phone calls using pre-written call scripts [1]. Effective secret shopper studies should clearly define a primary outcome variable and callers should change only one variable at a time when communicating with healthcare providers. For example, if assessing access to care based on insurance type, a simulated caller should define their outcome variable as: acceptance of Medicaid (Y/N); the only variable that should change between the two calls is the simulated patient’s type of insurance. Researchers should also consider nuances that impact these variables: a study comparing a variety of different insurance providers, for example, should maintain the same panel of physicians and benefit structures and only alter the insurance provider itself [6].

Beyond their primary outcome variable, researchers are able to collect additional variables of interest. For example, when assessing access to care, it may be helpful to record demographic information about a health center’s classification (e.g., academic vs. private vs. federally qualified health center). Callers can attempt to verify that this label is accurate during the call itself, as some websites may mischaracterize the nature of the practice [7]. Investigators can also consider recording the triage patterns within the center (e.g., if the patient is transferred to a physician, nurse practitioner, physician associate, etc.); additional requirements necessary to schedule an appointment (e.g., referrals, insurance acceptance/authorization, initial phone visit prior to in-person, etc.); cash price for a visit without insurance; and whether the prospective patient is referred out, and the address of the referral site.

### Developing a call script

Given that secret shoppers are most useful for auditing how providers and staff actually behave (and not how they claim they would), callers should inquire about a real (simulated) scenario rather than a hypothetical one. For example, the caller may pose as a patient experiencing symptoms of an illness and ask if they can schedule an appointment. This approach reveals more detailed information about clinic policies than would a study that simply asks which types of insurance an office would theoretically accept (which is more similar to administering a survey) [8]. Similarly, callers should not pre-screen offices to verify that the office accepts Medicaid prior to attempting to make an appointment, as doing so increases the likelihood of a sampling bias [9]. Therefore, callers should wait to bring up their insurance provider until after they begin the phone call to set up the appointment.

In addition, the call should accommodate the nuances that impact patients’ access to healthcare by asking probing questions and recording detailed information. For example, beyond determining whether the simulated patient is able to successfully schedule an appointment, studies can examine triage patterns to understand prioritization within a given clinic based on the patient’s insurance. This can be accomplished by several secret shoppers suggesting private vs. Medicaid insurance providers, with consistent presenting symptoms and patient history. Simulated patients can also identify other barriers to successful healthcare access, such as long appointment wait times, large upfront costs for Medicaid patients, or requirements for primary care provider referrals. If the practice is unable to provide care and refers the simulated patient to another provider (such as an emergency department), secret shoppers can ask for the address of that facility. This referral pattern information will help investigators understand shifts in healthcare service utilization. It will also provide insight into the geospatial distance patients must travel to seek healthcare services if they are referred to another site that accepts their insurance, which may indirectly pose another insurance-based barrier to ultimately accessing care. Likewise, the study can be designed to account for factors like state-level differences in Medicaid policies.

After developing a script with these factors in mind, investigators should pilot the calls by contacting real practices. Doing so will identify unanticipated scenarios, highlight potential nuances to probe, and facilitate the development of standardized responses to common questions. When refining the call script to seek relevant details, researchers should also be mindful of the efficiency of the calls and the plausibility of the scenario. Rather than running through a pre-determined list of questions, callers should endeavor to maintain a conversational tone and consult a call tree to focus their follow-up questions based on information they have already obtained (Table 1). This approach will both encourage the practice staff answering the phone and also mitigate any ethical concerns that secret shoppers divert time and resources away from real patients [10].

**Table 1:** Secret Shopper Best Practices Checklist

Developing a call script	Recording & reporting data	Making calls
• Seek IRB exemption • Define the primary outcome variable • Change only one variable at a time • Inquire about a real scenario • Don’t pre-screen offices to ensure that they accept Medicaid • Include questions that probe for nuances at individual practices and nationally • Pilot and refine the call script • Prioritize efficiency and plausibility	• Consider the health policy implications of data reported • Verify that demographic information about a practice is accurate • Record details that are relevant to the specifics of the research question and consider including additional demographic information and analytical tools to describe a practice within a state context	• Train callers in delivering standardized responses and periodically monitor their calls • Space out calls at the same practice by at least two weeks and consider calling at a different time on a different day of the week • Avoid centralized phone numbers of operating systems

### Making the calls

To ensure reproducibility, investigators should train callers to deliver standardized responses and should also supervise or periodically monitor the calls [11]. They should educate callers to understand the basics of their condition of interest, including descriptions of symptoms and relevant medical terminology [1]. Depending on the details of the scenario, researchers might consider taking additional steps to increase the perceived authenticity of the simulated patients. For example, one study noted that it employed single, middle-aged black men to pose as patients, which was consistent with the specific demographics of post-ACA expansion newly-insured residents of the location in which the study was conducted [7].

When making the calls, secret shoppers should take basic privacy precautions such as blocking phone numbers. Additionally, callers should be wary of centralized phone numbers or numbers that direct callers to the same operator (both of which may be used by a regional practice group with multiple satellite locations). If an operator or clinic staff member detects that the caller has contacted them several times or asks if the caller is conducting a survey, callers can respond, “My apologies, I’m trying to call a number of offices in the area to get more information,” and thank them for their time (Table 1).

### Ethical procedures

Many secret shopper studies will be eligible for exemption through an Institutional Review Board (IRB). An ethical analysis of simulated patient techniques in healthcare research emphasizes that the net risks to subjects involved in the study are outweighed by the benefit of monitoring healthcare access with a scientifically rigorous methodology, particularly when other measurement options fail to capture outcomes accurately [12, 13].

When developing the research protocol, however, investigators must still outline their plan to mitigate any potential ethical issues. For example, secret shopper studies have been critiqued for taking time and resources away from real patients [10]. A properly designed study will not schedule any actual appointments that take medical professionals away from real patients. The investigators need to create an efficient call script to minimize time spent speaking with call center staff. Investigators should consult their IRBs about ensuring subject confidentiality. Additionally, secret shopper research should produce data with policy relevance so that the case captures a pressing public health concern, and is clinically actionable [7]. Finally, researchers must be cognizant of the implications of their findings and the avenues for disclosing them, especially if they record instances of unsafe or illegal behavior [14]. Although guidelines for using simulated patients who present at clinics in person (rather than on the phone) is beyond the scope of this article, other researchers have also discussed the specific concerns of those ethical scenarios [15].

### Sampling methodology and frequency of data collection

The geographic scope of the locations selected can be tailored with these questions in mind (e.g. examining clinics in one city or comparing those across states). If the sample does not include every clinic in a defined region, then researchers should attempt to randomize the locations based on relevant characteristics to avoid sampling bias. For example, they might use a database to randomly select the same number of clinics from each state in the United States. They can then assign every clinic in each state a numeric value, after which a random number generator can select 25 different numbers for each state. If a clinic’s phone number is disconnected or incorrect, a new clinic can be randomly selected and contacted.

Investigators likely will need to call each clinic multiple times, using a script in which they have changed only one variable at a time. The study design may employ a computer program that randomly generates a call list and ensures that calls are made > 14 days apart [11]. To lessen the potential bias of one staff member’s response—and to avoid arousing suspicion—callers should consider attempting to make their second call on a different weekday and at a different time (Table 1).

### Analysis and dissemination of results

After making the calls, researchers can also collect additional demographic information about specific practices (often available through online databases) and contextualize them within state-level data. Broadly, these categories might include location classification (e.g., urban/suburban/rural), whether the state has expanded Medicaid, accreditation status, and the median income of the practice’s zip code. In addition, state-specific factors, such as the state’s Medicaid reimbursement rate for a specific office visit or procedure, can be utilized in the analysis.

When reporting data, researchers should summarize their findings, discuss limitations, assess the strength of each outcome, and consider the relevance to providers and policymakers [16]. Researchers should report their primary outcome data (e.g., “appointment success/Medicaid acceptance rate”), as well as the number of attempted calls, the number of successful calls, and the time period over which calls were made. They should also present interesting findings from secondary variables. Further research is needed to determine best practices for performing power calculations that account for the variability in the secret shopper data collection process [3]. In addition to using statistical software to analyze covariate and predictors of patient access, researchers might consider other analytical tools to measure access to healthcare. For example, the geoinformation software ArcGIS can provide information about driving time and distance between a practice and a referral location, or between a practice and the closest academic medical center. Applying multiple methodologies can illuminate additional factors constituting barriers to healthcare (Table 1).

## Discussion

The secret shopper methodology can be broadly applied to many healthcare settings. One particularly potent use of secret shopper methodologies is as a mechanism to assess the delivery of healthcare. For example, family medicine, with its emphasis on preventative healthcare—which at its core has the aims to provide equitable care to all—represents one ideal opportunity to assess and improve healthcare delivery, elevate the patient experience, and reduce disparities in access. The methodology outlined here can be used to ascertain areas of potential improvement within the healthcare cascade [17, 18].

It is challenging to objectively measure patients’ ability to access healthcare. When surveyed about appointment availability, physicians’ offices tend to overestimate their capacity to accept hypothetical new patients [19]. Patient and physician surveys often fail to accurately capture biases—particularly those related to sensitive or stigmatized topics—which may impact patients’ experiences with healthcare providers and, consequently, their access to appropriate care [19]. Furthermore, physicians’ offices may not be forthcoming about their health insurance policies, especially when discussing their ability or willingness to promptly accept Medicaid patients [19]. Each of these elements is integral to providing good care in family medicine.

The secret shopper methodology serves as an objective measure for studies seeking to evaluate patient access to care [20]. This method is particularly useful in overcoming the “Hawthorne effect” in situations similar to the one described above, in which providers’ behaviors or policies might change if they were aware that they were being observed [21]. For example, an audit of orthopedic surgery practices to evaluate patient access to knee and primary and revision arthroplasty found that orthopedic surgeons’ offices responded differently to a faxed survey about Medicaid acceptance policies than they did to a simulated Medicaid patient calling and attempting to schedule an appointment [22]. A study examining delays to emergent surgical care revealed discrepancies in emergency department referrals based on insurance status [23]. These results suggest that practice policies and staff behavior may not be accurately captured in a survey. Therefore, the results of these studies can be used to enhance patient access to care and the quality of care they receive.

Insurance-based discrepancies in access to care impact the healthcare system in a variety of ways. Understanding shifts in the burden of healthcare service utilization can reveal gaps between policy and practice. Accordingly, the United States Department of Health and Human Services recommends using secret shoppers to measure healthcare access [24]. A 1994 study by the Medicaid Access Study Group employed this methodology and found that Medicaid patients were less able to access timely medical appointments, a finding that contributed to the explanation for that population’s increased use of emergency departments for nonurgent issues [25]. Other secret shopper studies have revealed insurance-related disparities in access to primary [8, 18, 26–28], follow-up [11], orthopedic [29], dermatologic [30], pediatric [31, 32], newborn [33], reproductive [5], and psychiatric [34] care, among others [35]. In addition to highlighting challenges associated with insurance, secret shopper studies can assess administrative staff’s knowledge of details related to coverage, such as the likelihood of receiving a surprise bill or the availability of alternative payment options [36, 37].

Furthermore, simulated patients can obtain more detailed information about the intricacies of patients’ experiences within a complex and evolving healthcare system. The last decade has seen substantial increases in vertical and horizontal consolidation of health organizations and medical practices [38, 39]. These consolidations have affected all sectors, but particularly family medicine. To cite a few downstream effects: increased costs, increased travel time for patients, and no improvement in quality of care [40]. These mergers are often associated with higher prices and spending, and budgeting decisions inevitably affect reimbursement, clinical decision making, local competition, referrals, and patient experiences [41–43]. These nuances impact access to care but might not be apparent on practice websites or in healthcare policy. For example, in addition to determining appointment availability based on insurance type, secret shopper studies can probe for information about how patients are treated by providers or staff based on their insurance. Secret shoppers may also identify additional barriers to receiving care, such as referral requirements, long wait times before obtaining appointments, and the requirement to send records and obtain testing results prior to the visit, which might not be expected of patients with private insurance [22].

However, it is important to also address the challenges of deploying these studies. Secret shopper studies need to be done systematically or can be subject to bias. It can be difficult to achieve large enough numbers to power studies, so careful considerations need to be taken to begin with a broad targeted cohort. There is also a subjective nature of data acquisition that is challenging to control. Iterative processes should be undertaken, when possible, as the role of discrimination vs. human emotions needs to be teased apart. Additionally, once providers learn of the secret shoppers, this can perhaps engender distrust on the part of the healthcare providers being assessed, which may damage any future relations. Therefore, it is important that researchers of this methodology, when publishing and sharing their results, do not identify specific entities for privacy reasons given that these entities never gave consent to participate.

These findings are especially useful for evaluating access to care in a period following Medicaid expansion and the COVID-19 pandemic in the United States, when policymakers, providers, and patients alike seek to understand whether health insurance coverage alleviates differences in healthcare access to the underinsured. Secret shopper studies are well-positioned to expose these disparities and provide insight into how patients navigate the healthcare system [44].

### Case study

### Examining access to urgent care based on insurance type

This study sought to examine insurance-related disparities in access to care at urgent care centers in the United States. We designed a secret shopper study to assess whether callers posing as simulated patients with either private insurance or Medicaid were able to successfully schedule an appointment. Below, we analyze our process in accordance with the best practices outlined.

#### 1. Seek IRB exemption

Our study design received an institutional review board exemption from our institution’s IRB.

#### 2. Define the primary outcome variable

Our primary outcome variable was acceptance of Medicaid.

#### *3. Change only onevariable at a time*

Our callers called once asking about a patient paying with private (Blue Cross) insurance and once about paying with Medicaid. All other details remained the same.

#### *4. Inquire about a real scenario*

Our callers explained that they were inquiring on behalf of their father, who had been previously diagnosed with an incarcerated inguinal hernia and was currently experiencing symptoms of strangulation. They stated his insurance type and asked if they could bring him into the urgent care center.

#### 5. Don’t pre-screen offices to ensure that they accept Medicaid

We used a random number generator to select 25 urgent care centers to call per state, and did not pre-screen any of them to verify that they accepted Medicaid.

#### 6. Include questions that probe for nuances

Our call script included a variety of follow-up questions after we presented the initial scenario: “What kind of provider would he be seeing (doctor, nurse, PA)?” “What would the wait time be? Do you have a self-pay policy for uninsured patients?” “Are there any discounts?” “Do you know the maximum price you would have to pay?” “What’s the closest Emergency Department to your office?” These questions allowed us to capture a more accurate understanding of a patient’s experience attempting to access care.

#### 7. Pilot and refine the call script (and prioritize plausibility and efficiency)

Prior to beginning the data collection, we piloted the script by contacting several offices. We adjusted our scripts accordingly to streamline the conversation and ask more relevant questions.

#### 8. Consider the health policy implications of data reported

We knew that the urgent care industry had experienced astronomical growth in recent years, but we wanted to determine the degree to which urgent care is accessible and how it impacts the care of surgical conditions. Collecting and reporting this data has important health policy implications.

#### 9. Verify that the practice type is categorized correctly

When we called, we specified that we were seeking an urgent care center and excluded the center from our results if the office said they did not meet our criteria.

*10. Record details that are relevant to the research objective; consider including additional demographic information and analytical tools to describe a practice within a state context*.

We recorded data describing both individual centers and entire states. Our categories included: urgent care center classification (independent, private practice, primary care, health network, academic); accreditation status; zip code median income; driving distance from an academic medical center; state Medicaid expansion status and reimbursement level for a new patient visit; and state population size. We analyzed our data using JMP Pro. We also identified urgent care centers within a 5-mile driving radius of an academic medical center using the ArcGIS Mapping System.

We presented a univariate and multivariate analysis of center-specific and community-level urgent care center characteristics. We concluded with relevant policy findings about predictors of Medicaid acceptance, suggested that urgent care centers may be referring Medicaid patients to safety-net hospitals. We urged future investigators to delve deeper into understanding the impact of urgent care centers on referral rates and patterns, healthcare disparities, and delays to receiving appropriate care.

#### 11. Train callers in delivering standardized responses and periodically monitor their calls

We trained callers and provided feedback on several practice calls before they began calling sites independently.

#### 12. Block phone numbers

When possible, we blocked our numbers. Notably, several urgent care practice offices did not accept calls from a blocked number, so we made sure to call from a different phone for the second call.

#### 13. Space out calls

We called practices at least > 14 days apart and, whenever possible, from a different phone number and on a different weekday and time.

#### 14. Avoid centralized phone numbers of operating systems

When we encountered a centralized phone system, we attempted to look online for direct extensions that would lead us to individual centers instead. If an operator realized that we had called multiple times, we explained that we were trying to get a better sense of regional healthcare options for the patient (the simulated caller’s father).

#### 15. Be on the lookout for aberrances or unusual activity

Those making the phone calls must make each call with an eye open for anomalous or peculiar results. We have commonly been surprised by some of the answers given to us by the operators, and at times have uncovered findings that led us to alter our study design or propose a new study to probe a different question.

## Data Availability

N/A.
